# {2,2′-[(2,2-Dimethyl­propane-1,3-di­yl)­bis­(nitrilo­methyl­idyne)]­diphenolato}­dioxidomolybdenum(VI)

**DOI:** 10.1107/S160053680802182X

**Published:** 2008-07-19

**Authors:** Alireza Abbasi, Iran Sheikhshoaie, Abbas Saghaei, Niaz Monadi

**Affiliations:** a School of Chemistry, University College of Science, University of Tehran, Iran; b Chemistry Department, Shahid Bahonar University, Kerman, Iran; c Islamic Azad University, Science and Research Branch, Tehran, Iran

## Abstract

In the structure of the title compound, [Mo(C_19_H_20_N_2_O_2_)O_2_], the Mo atom exhibits oxidation state +VI and is surrounded by two O atoms and the tetra­dentate Schiff base ligand 2,2′-[(2,2-dimethyl­propane-1,3-di­yl)bis­(nitrilo­methyl­idyne)]diphenolate in a distorted octa­hedral configuration. An intra­molecular C—H⋯O hydrogen bond between a methyl­ene group and one O atom of the O=Mo^VI^=O unit, as well as additional inter­molecular hydrogen bonds between neighboring mol­ecules, lead to a weakly bonded inversion-symmetric dimeric structure.

## Related literature

For related structures with O=Mo^VI^=O units and for synthesis, see: Arnaiz *et al.* (2000 ▶); Holm *et al.* (1996 ▶); Syamal & Maurya (1989 ▶). The crystal structure of the free ligand *N*,*N′*-bis­(2-hydroxy­benzyl­idene)-2,2-dimethyl-1,3-propane­diamine was described by Corden *et al.* (1996 ▶).
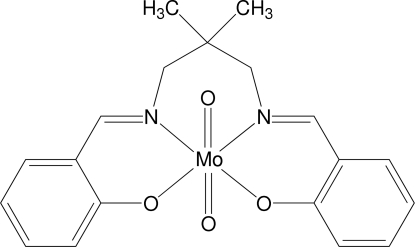


## Experimental

### 

#### Crystal data

[Mo(C_19_H_20_N_2_O_2_)O_2_]
*M* *_r_* = 436.31
Triclinic, 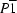

*a* = 9.3875 (10) Å
*b* = 9.5597 (10) Å
*c* = 11.0422 (11) Å
α = 104.6790 (17)°
β = 108.1939 (17)°
γ = 101.1218 (17)°
*V* = 869.87 (16) Å^3^
*Z* = 2
Mo *K*α radiation
μ = 0.78 mm^−1^
*T* = 100 (2) K
0.18 × 0.12 × 0.06 mm

#### Data collection

Bruker APEXII CCD diffractometer
Absorption correction: none
8420 measured reflections
3413 independent reflections
3204 reflections with *I* > 2σ(*I*)
*R*_int_ = 0.020

#### Refinement

*R*[*F* ^2^ > 2σ(*F* ^2^)] = 0.021
*wR*(*F* ^2^) = 0.051
*S* = 1.07
3413 reflections
237 parameters
H-atom parameters constrained
Δρ_max_ = 0.42 e Å^−3^
Δρ_min_ = −0.53 e Å^−3^

### 

Data collection: *APEX2* (Bruker, 2005 ▶); cell refinement: *APEX2*; data reduction: *APEX2*; program(s) used to solve structure: *SHELXS97* (Sheldrick, 2008 ▶); program(s) used to refine structure: *SHELXL97* (Sheldrick, 2008 ▶); molecular graphics: *DIAMOND* (Brandenburg, 2001 ▶); software used to prepare material for publication: *PLATON* (Spek, 2003 ▶).

## Supplementary Material

Crystal structure: contains datablocks I, global. DOI: 10.1107/S160053680802182X/wm2183sup1.cif

Structure factors: contains datablocks I. DOI: 10.1107/S160053680802182X/wm2183Isup2.hkl

Additional supplementary materials:  crystallographic information; 3D view; checkCIF report

## Figures and Tables

**Table 1:** Selected bond lengths (Å)

Mo1—O1	1.7072 (14)
Mo1—O2	1.7120 (14)
Mo1—O4	1.9373 (13)
Mo1—O3	2.0917 (14)
Mo1—N2	2.1442 (16)
Mo1—N1	2.3402 (16)

**Table 2:** Hydrogen-bond geometry (Å, °)

*D*—H⋯*A*	*D*—H	H⋯*A*	*D*⋯*A*	*D*—H⋯*A*
C4—H4*A*⋯O2	0.97	2.49	2.986 (2)	112
C1—H1⋯O1^i^	0.93	2.54	3.227 (2)	131
C4—H4*B*⋯O1^i^	0.97	2.56	3.294 (2)	132
C7—H7⋯O2^i^	0.93	2.55	3.315 (2)	140

Symmetry code: (i) 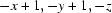.

**Fig. 1.**
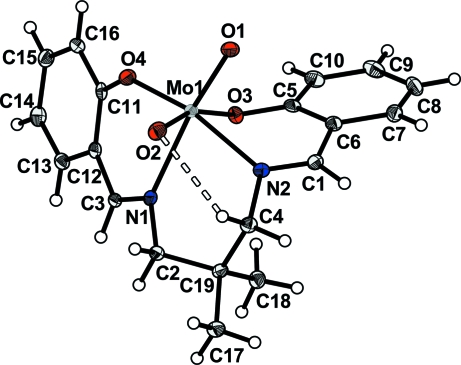
Molecular structure of (I), with displacement ellipsoids drawn at the 50% probability level. H atoms are shown as circles of arbitrary radius. Hydrogen bonds are represented as dotted lines.

**Fig. 2.**
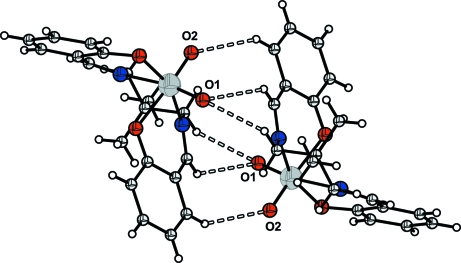
The inversion-symmetric dimeric structure of (I). Hydrogen bonds are represented as dotted lines.

**Fig. 3.**
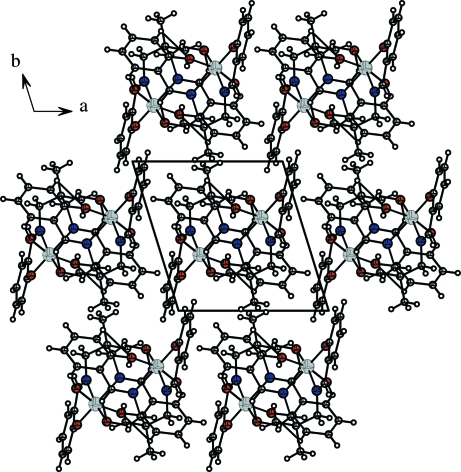
Packing plot of (I) along [001].

## supplementary crystallographic information

### Comment

Numerous chemical reactions are catalyzed by compounds containing complexes with the dioxidomolybdenum(VI) unit O═Mo^VI^═O (Arnaiz *et al.* 2000). Moreover, Schiff base compounds containing molybdenum play a significant role in the chemistry of molybdoenzymes (Holm *et al.* 1996; Syamal & Maurya, 1989). Therefore we are interested in the structural chemistry of dioxidomolybdenum(VI) complexes and have synthesized and structurally characterized the title compound, MoO_2_(C_19_H_20_N_2_O_2_), (I).

The molecular structure of (I) and the atom-numbering scheme are shown in Fig. 1. The molybdenum(VI) atom is in a distorted octahedral coordination by two oxygen atoms and one tetradentate ligand *L*, where *L* is *N*,*N'*-bis(2-hydroxybenzylidene)-2,2-dimethyl-1,3-propanediamine. The Mo–O distances of the oxido ligands are significantly shorter (average 1.71 Å) than the corresponding distances to the O atoms of the tetradentate ligand (average 2.02 Å). The Mo–N distances are the longest (average 2.24 Å). An intramolecular hydrogen bond is present between the methylene group and one O atom from the O═Mo═O group (C4—H41···O2, 2.985 Å). Much weaker intermolecular hydrogen bonds exist between neighboring molecules, leading to an dimer structure (see hydrogen bond Table and Fig. 2). For a packing plot of the structure, see Fig. 3. Resulting from the coordination of the tetradentate *L* ligand to the molybdenum ion, the chelate ligand is more twisted than the free ligand, with a larger dihedral angle between two phenyl rings of 75.2 (1)° compared to the free ligand with 68.7 (1)° (Corden *et al.*,  1996).

### Experimental

The title compound was prepared by adding MoO_2_(acac)_2_ and the ligand H_2_*L* (Corden *et al.*, 1996) in the molar ratio 1:1 to 30 mL dry methanol, followed by refluxing the solution for 1 h. Small prismatic, yellowish crystals precipitated which were filtered off and recrystallized from acetonitrile to get a better crystal quality.

### Refinement

All H atoms were placed in calculated positions with C—H = 0.93 Å for H atoms bonded to sp^2^ C atoms, and C—H = 0.97 Å for H atoms bonded to sp^3^ C atoms, and constrained to ride on their parent atoms with *U*_iso_(H) = 1.2 and 1.5 *U*_eq_(C), respectively.

### Crystal data

**Table:**

[Mo(C_19_H_20_N_2_O_2_)O_2_]	*Z* = 2
*M**_r_* = 436.31	*F*_000_ = 444
Triclinic, *P*1	*D*_x_ = 1.666 Mg m^−^^3^
Hall symbol: -P 1	Mo *K*α radiation λ = 0.71073 Å
*a* = 9.3875 (10) Å	Cell parameters from 8420 reflections
*b* = 9.5597 (10) Å	θ = 2.5–26.0º
*c* = 11.0422 (11) Å	µ = 0.78 mm^−^^1^
α = 104.6790 (17)º	*T* = 100 (2) K
β = 108.1939 (17)º	Prism, light yellow
γ = 101.1218 (17)º	0.18 × 0.12 × 0.06 mm
*V* = 869.87 (16) Å^3^	

### Data collection

**Table:**

Bruker APEXII CCD diffractometer	3204 reflections with *I* > 2σ(*I*)
Radiation source: fine-focus sealed tube	*R*_int_ = 0.020
Monochromator: graphite	θ_max_ = 26.0º
*T* = 100(2) K	θ_min_ = 2.1º
ω scans	*h* = −11→11
Absorption correction: none	*k* = −11→11
8420 measured reflections	*l* = −13→13
3413 independent reflections	

### Refinement

**Table:**

Refinement on *F*^2^	Secondary atom site location: difference Fourier map
Least-squares matrix: full	Hydrogen site location: inferred from neighbouring sites
*R*[*F*^2^ > 2σ(*F*^2^)] = 0.021	H-atom parameters constrained
*wR*(*F*^2^) = 0.051	*w* = 1/[σ^2^(*F*_o_^2^) + (0.0184*P*)^2^ + 0.8169*P*] where *P* = (*F*_o_^2^ + 2*F*_c_^2^)/3
*S* = 1.07	(Δ/σ)_max_ = 0.001
3413 reflections	Δρ_max_ = 0.42 e Å^−^^3^
237 parameters	Δρ_min_ = −0.53 e Å^−^^3^
Primary atom site location: structure-invariant direct methods	Extinction correction: none

### Special details

**Table:**

Geometry. All e.s.d.'s (except the e.s.d. in the dihedral angle between two l.s. planes) are estimated using the full covariance matrix. The cell e.s.d.'s are taken into account individually in the estimation of e.s.d.'s in distances, angles and torsion angles; correlations between e.s.d.'s in cell parameters are only used when they are defined by crystal symmetry. An approximate (isotropic) treatment of cell e.s.d.'s is used for estimating e.s.d.'s involving l.s. planes.
Refinement. Refinement of *F*^2^ against ALL reflections. The weighted *R*-factor *wR* and goodness of fit *S* are based on *F*^2^, conventional *R*-factors *R* are based on *F*, with *F* set to zero for negative *F*^2^. The threshold expression of *F*^2^ > σ(*F*^2^) is used only for calculating *R*-factors(gt) *etc*. and is not relevant to the choice of reflections for refinement. *R*-factors based on *F*^2^ are statistically about twice as large as those based on *F*, and *R*- factors based on ALL data will be even larger.

### Fractional atomic coordinates and isotropic or equivalent isotropic displacement parameters (Å^2^)

**Table:**

	*x*	*y*	*z*	*U*_iso_*/*U*_eq_	
Mo1	0.742950 (19)	0.625366 (18)	0.282813 (16)	0.01031 (6)	
O1	0.70502 (16)	0.74204 (15)	0.19043 (14)	0.0161 (3)	
O2	0.84039 (16)	0.52119 (16)	0.20730 (14)	0.0167 (3)	
O3	0.59172 (16)	0.68583 (15)	0.37734 (13)	0.0135 (3)	
O4	0.90918 (16)	0.76348 (15)	0.44982 (13)	0.0146 (3)	
N1	0.75678 (18)	0.46531 (18)	0.41243 (16)	0.0115 (3)	
N2	0.52861 (19)	0.44879 (18)	0.15515 (16)	0.0112 (3)	
C1	0.3927 (2)	0.4737 (2)	0.11735 (19)	0.0127 (4)	
H1	0.3111	0.3981	0.0443	0.015*	
C2	0.7108 (2)	0.3002 (2)	0.34369 (19)	0.0122 (4)	
H2A	0.7190	0.2512	0.4115	0.015*	
H2B	0.7855	0.2782	0.3032	0.015*	
C3	0.8231 (2)	0.5116 (2)	0.5423 (2)	0.0121 (4)	
H3	0.8229	0.4382	0.5838	0.014*	
C4	0.5301 (2)	0.2902 (2)	0.11404 (19)	0.0131 (4)	
H4A	0.6177	0.2836	0.0863	0.016*	
H4B	0.4339	0.2289	0.0375	0.016*	
C5	0.4553 (2)	0.7013 (2)	0.3131 (2)	0.0127 (4)	
C6	0.3575 (2)	0.6066 (2)	0.1779 (2)	0.0132 (4)	
C7	0.2101 (2)	0.6264 (2)	0.1145 (2)	0.0156 (4)	
H7	0.1467	0.5643	0.0262	0.019*	
C8	0.1596 (2)	0.7357 (2)	0.1811 (2)	0.0188 (4)	
H8	0.0631	0.7486	0.1380	0.023*	
C9	0.2546 (3)	0.8278 (2)	0.3146 (2)	0.0189 (4)	
H9	0.2204	0.9020	0.3600	0.023*	
C10	0.3979 (2)	0.8102 (2)	0.3795 (2)	0.0161 (4)	
H10	0.4579	0.8713	0.4688	0.019*	
C11	0.9390 (2)	0.7870 (2)	0.58181 (19)	0.0122 (4)	
C12	0.8984 (2)	0.6685 (2)	0.62999 (19)	0.0120 (4)	
C13	0.9480 (2)	0.6997 (2)	0.7706 (2)	0.0139 (4)	
H13	0.9255	0.6212	0.8035	0.017*	
C14	1.0294 (2)	0.8440 (2)	0.8609 (2)	0.0154 (4)	
H14	1.0595	0.8633	0.9536	0.019*	
C15	1.0658 (2)	0.9608 (2)	0.8110 (2)	0.0156 (4)	
H15	1.1204	1.0585	0.8712	0.019*	
C16	1.0218 (2)	0.9330 (2)	0.6732 (2)	0.0143 (4)	
H16	1.0473	1.0118	0.6414	0.017*	
C17	0.5230 (2)	0.0591 (2)	0.1785 (2)	0.0166 (4)	
H17A	0.5356	0.0183	0.2509	0.025*	
H17B	0.5999	0.0428	0.1404	0.025*	
H17C	0.4197	0.0095	0.1098	0.025*	
C18	0.4199 (2)	0.2557 (2)	0.2899 (2)	0.0156 (4)	
H18A	0.3185	0.2163	0.2174	0.023*	
H18B	0.4408	0.3623	0.3329	0.023*	
H18C	0.4216	0.2051	0.3549	0.023*	
C19	0.5451 (2)	0.2294 (2)	0.23343 (19)	0.0119 (4)	

### Atomic displacement parameters (Å^2^)

**Table:**

	*U*^11^	*U*^22^	*U*^33^	*U*^12^	*U*^13^	*U*^23^
Mo1	0.01067 (9)	0.01016 (9)	0.00976 (9)	0.00212 (6)	0.00322 (6)	0.00433 (6)
O1	0.0185 (7)	0.0120 (7)	0.0142 (7)	0.0015 (6)	0.0028 (6)	0.0051 (6)
O2	0.0143 (7)	0.0182 (7)	0.0193 (7)	0.0037 (6)	0.0090 (6)	0.0064 (6)
O3	0.0135 (7)	0.0145 (7)	0.0121 (7)	0.0054 (6)	0.0038 (5)	0.0046 (5)
O4	0.0148 (7)	0.0147 (7)	0.0117 (7)	0.0006 (6)	0.0026 (6)	0.0058 (6)
N1	0.0081 (8)	0.0116 (8)	0.0149 (8)	0.0030 (6)	0.0043 (6)	0.0046 (7)
N2	0.0139 (8)	0.0106 (8)	0.0093 (8)	0.0031 (6)	0.0052 (6)	0.0032 (6)
C1	0.0132 (10)	0.0134 (9)	0.0099 (9)	0.0013 (8)	0.0031 (7)	0.0047 (7)
C2	0.0116 (9)	0.0116 (9)	0.0150 (10)	0.0042 (8)	0.0053 (8)	0.0059 (8)
C3	0.0086 (9)	0.0135 (9)	0.0167 (10)	0.0037 (7)	0.0048 (8)	0.0090 (8)
C4	0.0152 (10)	0.0106 (9)	0.0114 (9)	0.0030 (8)	0.0045 (8)	0.0014 (7)
C5	0.0145 (10)	0.0123 (9)	0.0150 (10)	0.0036 (8)	0.0069 (8)	0.0088 (8)
C6	0.0137 (10)	0.0142 (9)	0.0148 (10)	0.0041 (8)	0.0070 (8)	0.0082 (8)
C7	0.0133 (10)	0.0184 (10)	0.0157 (10)	0.0035 (8)	0.0047 (8)	0.0083 (8)
C8	0.0161 (10)	0.0238 (11)	0.0253 (11)	0.0111 (9)	0.0100 (9)	0.0163 (9)
C9	0.0248 (12)	0.0181 (10)	0.0244 (11)	0.0123 (9)	0.0156 (9)	0.0121 (9)
C10	0.0220 (11)	0.0143 (10)	0.0141 (10)	0.0066 (8)	0.0084 (8)	0.0058 (8)
C11	0.0075 (9)	0.0169 (10)	0.0124 (9)	0.0052 (8)	0.0023 (7)	0.0059 (8)
C12	0.0078 (9)	0.0142 (9)	0.0148 (10)	0.0044 (7)	0.0041 (7)	0.0056 (8)
C13	0.0119 (10)	0.0173 (10)	0.0163 (10)	0.0060 (8)	0.0068 (8)	0.0091 (8)
C14	0.0140 (10)	0.0207 (10)	0.0110 (9)	0.0054 (8)	0.0045 (8)	0.0046 (8)
C15	0.0129 (10)	0.0139 (10)	0.0160 (10)	0.0035 (8)	0.0032 (8)	0.0019 (8)
C16	0.0127 (10)	0.0131 (10)	0.0181 (10)	0.0044 (8)	0.0043 (8)	0.0083 (8)
C17	0.0173 (10)	0.0122 (10)	0.0175 (10)	0.0026 (8)	0.0047 (8)	0.0042 (8)
C18	0.0131 (10)	0.0190 (10)	0.0168 (10)	0.0049 (8)	0.0067 (8)	0.0083 (8)
C19	0.0117 (9)	0.0098 (9)	0.0139 (9)	0.0021 (7)	0.0054 (8)	0.0035 (8)

### Geometric parameters (Å, °)

**Table:**

Mo1—O1	1.7072 (14)	C7—C8	1.370 (3)
Mo1—O2	1.7120 (14)	C7—H7	0.9300
Mo1—O4	1.9373 (13)	C8—C9	1.401 (3)
Mo1—O3	2.0917 (14)	C8—H8	0.9300
Mo1—N2	2.1442 (16)	C9—C10	1.378 (3)
Mo1—N1	2.3402 (16)	C9—H9	0.9300
O3—C5	1.313 (2)	C10—H10	0.9300
O4—C11	1.345 (2)	C11—C16	1.395 (3)
N1—C3	1.286 (2)	C11—C12	1.407 (3)
N1—C2	1.477 (2)	C12—C13	1.406 (3)
N2—C1	1.301 (3)	C13—C14	1.379 (3)
N2—C4	1.472 (2)	C13—H13	0.9300
C1—C6	1.428 (3)	C14—C15	1.395 (3)
C1—H1	0.9300	C14—H14	0.9300
C2—C19	1.534 (3)	C15—C16	1.385 (3)
C2—H2A	0.9700	C15—H15	0.9300
C2—H2B	0.9700	C16—H16	0.9300
C3—C12	1.454 (3)	C17—C19	1.534 (3)
C3—H3	0.9300	C17—H17A	0.9600
C4—C19	1.549 (3)	C17—H17B	0.9600
C4—H4A	0.9700	C17—H17C	0.9600
C4—H4B	0.9700	C18—C19	1.525 (3)
C5—C10	1.410 (3)	C18—H18A	0.9600
C5—C6	1.425 (3)	C18—H18B	0.9600
C6—C7	1.415 (3)	C18—H18C	0.9600
			
O1—Mo1—O2	103.33 (7)	C8—C7—H7	119.5
O1—Mo1—O4	101.99 (6)	C6—C7—H7	119.5
O2—Mo1—O4	102.79 (6)	C7—C8—C9	119.36 (19)
O1—Mo1—O3	90.82 (6)	C7—C8—H8	120.3
O2—Mo1—O3	161.79 (6)	C9—C8—H8	120.3
O4—Mo1—O3	85.05 (6)	C10—C9—C8	120.94 (19)
O1—Mo1—N2	93.87 (6)	C10—C9—H9	119.5
O2—Mo1—N2	88.54 (6)	C8—C9—H9	119.5
O4—Mo1—N2	157.64 (6)	C9—C10—C5	121.24 (19)
O3—Mo1—N2	79.01 (6)	C9—C10—H10	119.4
O1—Mo1—N1	170.94 (6)	C5—C10—H10	119.4
O2—Mo1—N1	84.17 (6)	O4—C11—C16	117.93 (17)
O4—Mo1—N1	80.95 (6)	O4—C11—C12	122.15 (17)
O3—Mo1—N1	80.84 (5)	C16—C11—C12	119.80 (18)
N2—Mo1—N1	81.12 (6)	C13—C12—C11	118.62 (18)
C5—O3—Mo1	123.46 (12)	C13—C12—C3	117.94 (17)
C11—O4—Mo1	134.29 (12)	C11—C12—C3	123.05 (17)
C3—N1—C2	115.90 (16)	C14—C13—C12	121.52 (18)
C3—N1—Mo1	124.12 (13)	C14—C13—H13	119.2
C2—N1—Mo1	119.23 (12)	C12—C13—H13	119.2
C1—N2—C4	117.18 (16)	C13—C14—C15	118.99 (18)
C1—N2—Mo1	122.83 (13)	C13—C14—H14	120.5
C4—N2—Mo1	119.87 (12)	C15—C14—H14	120.5
N2—C1—C6	125.94 (18)	C16—C15—C14	120.85 (19)
N2—C1—H1	117.0	C16—C15—H15	119.6
C6—C1—H1	117.0	C14—C15—H15	119.6
N1—C2—C19	115.73 (15)	C15—C16—C11	120.17 (18)
N1—C2—H2A	108.3	C15—C16—H16	119.9
C19—C2—H2A	108.3	C11—C16—H16	119.9
N1—C2—H2B	108.3	C19—C17—H17A	109.5
C19—C2—H2B	108.3	C19—C17—H17B	109.5
H2A—C2—H2B	107.4	H17A—C17—H17B	109.5
N1—C3—C12	125.78 (17)	C19—C17—H17C	109.5
N1—C3—H3	117.1	H17A—C17—H17C	109.5
C12—C3—H3	117.1	H17B—C17—H17C	109.5
N2—C4—C19	110.04 (15)	C19—C18—H18A	109.5
N2—C4—H4A	109.7	C19—C18—H18B	109.5
C19—C4—H4A	109.7	H18A—C18—H18B	109.5
N2—C4—H4B	109.7	C19—C18—H18C	109.5
C19—C4—H4B	109.7	H18A—C18—H18C	109.5
H4A—C4—H4B	108.2	H18B—C18—H18C	109.5
O3—C5—C10	120.15 (18)	C18—C19—C2	111.61 (16)
O3—C5—C6	122.19 (18)	C18—C19—C17	109.71 (16)
C10—C5—C6	117.62 (18)	C2—C19—C17	106.47 (16)
C7—C6—C5	119.77 (18)	C18—C19—C4	110.70 (16)
C7—C6—C1	119.37 (18)	C2—C19—C4	110.88 (15)
C5—C6—C1	119.60 (17)	C17—C19—C4	107.29 (15)
C8—C7—C6	121.04 (19)		
			
O1—Mo1—O3—C5	41.55 (15)	Mo1—O3—C5—C10	−146.68 (14)
O2—Mo1—O3—C5	−99.9 (2)	Mo1—O3—C5—C6	35.9 (2)
O4—Mo1—O3—C5	143.51 (15)	O3—C5—C6—C7	178.78 (17)
N2—Mo1—O3—C5	−52.23 (14)	C10—C5—C6—C7	1.3 (3)
N1—Mo1—O3—C5	−134.88 (15)	O3—C5—C6—C1	11.6 (3)
O1—Mo1—O4—C11	135.68 (17)	C10—C5—C6—C1	−165.83 (18)
O2—Mo1—O4—C11	−117.46 (17)	N2—C1—C6—C7	171.47 (18)
O3—Mo1—O4—C11	45.89 (17)	N2—C1—C6—C5	−21.3 (3)
N2—Mo1—O4—C11	1.4 (3)	C5—C6—C7—C8	0.1 (3)
N1—Mo1—O4—C11	−35.61 (17)	C1—C6—C7—C8	167.25 (19)
O2—Mo1—N1—C3	121.32 (16)	C6—C7—C8—C9	−0.8 (3)
O4—Mo1—N1—C3	17.34 (15)	C7—C8—C9—C10	0.1 (3)
O3—Mo1—N1—C3	−69.06 (15)	C8—C9—C10—C5	1.3 (3)
N2—Mo1—N1—C3	−149.25 (16)	O3—C5—C10—C9	−179.54 (18)
O2—Mo1—N1—C2	−48.30 (13)	C6—C5—C10—C9	−2.0 (3)
O4—Mo1—N1—C2	−152.29 (13)	Mo1—O4—C11—C16	−150.25 (15)
O3—Mo1—N1—C2	121.32 (13)	Mo1—O4—C11—C12	33.8 (3)
N2—Mo1—N1—C2	41.12 (13)	O4—C11—C12—C13	173.48 (17)
O1—Mo1—N2—C1	−48.07 (15)	C16—C11—C12—C13	−2.4 (3)
O2—Mo1—N2—C1	−151.34 (15)	O4—C11—C12—C3	0.8 (3)
O4—Mo1—N2—C1	87.3 (2)	C16—C11—C12—C3	−175.13 (18)
O3—Mo1—N2—C1	42.02 (15)	N1—C3—C12—C13	172.40 (18)
N1—Mo1—N2—C1	124.33 (15)	N1—C3—C12—C11	−14.9 (3)
O1—Mo1—N2—C4	136.02 (13)	C11—C12—C13—C14	2.7 (3)
O2—Mo1—N2—C4	32.75 (14)	C3—C12—C13—C14	175.75 (18)
O4—Mo1—N2—C4	−88.6 (2)	C12—C13—C14—C15	−1.4 (3)
O3—Mo1—N2—C4	−133.89 (14)	C13—C14—C15—C16	−0.2 (3)
N1—Mo1—N2—C4	−51.57 (13)	C14—C15—C16—C11	0.4 (3)
C4—N2—C1—C6	159.34 (18)	O4—C11—C16—C15	−175.12 (17)
Mo1—N2—C1—C6	−16.7 (3)	C12—C11—C16—C15	1.0 (3)
C3—N1—C2—C19	133.97 (18)	N1—C2—C19—C18	−59.5 (2)
Mo1—N1—C2—C19	−55.57 (19)	N1—C2—C19—C17	−179.21 (16)
C2—N1—C3—C12	170.57 (17)	N1—C2—C19—C4	64.4 (2)
Mo1—N1—C3—C12	0.6 (3)	N2—C4—C19—C18	52.6 (2)
C1—N2—C4—C19	−100.26 (19)	N2—C4—C19—C2	−71.82 (19)
Mo1—N2—C4—C19	75.87 (17)	N2—C4—C19—C17	172.30 (15)

### Hydrogen-bond geometry (Å, °)

**Table:**

*D*—H···*A*	*D*—H	H···*A*	*D*···*A*	*D*—H···*A*
C4—H4A···O2	0.97	2.49	2.986 (2)	112
C1—H1···O1^i^	0.93	2.54	3.227 (2)	131
C4—H4B···O1^i^	0.97	2.56	3.294 (2)	132
C7—H7···O2^i^	0.93	2.55	3.315 (2)	140

Symmetry codes: (i) −*x*+1, −*y*+1, −*z*.
